# Sex-Specific Expression of the X-Linked Histone Demethylase Gene *Jarid1c* in Brain

**DOI:** 10.1371/journal.pone.0002553

**Published:** 2008-07-02

**Authors:** Jun Xu, Xinxian Deng, Christine M. Disteche

**Affiliations:** 1 Department of Pathology, University of Washington, Seattle, Washington, United States of America; 2 Department of Biomedical Sciences, Tufts University Cummings School of Veterinary Medicine, North Grafton, Massachusetts, United States of America; 3 Department of Medicine, University of Washington, Seattle, Washington, United States of America; University of Massachusetts Medical School, United States of America

## Abstract

*Jarid1c*, an X-linked gene coding for a histone demethylase, plays an important role in brain development and function. Notably, *JARID1C* mutations cause mental retardation and increased aggression in humans. These phenotypes are consistent with the expression patterns we have identified in mouse brain where *Jarid1c* mRNA was detected in hippocampus, hypothalamus, and cerebellum. *Jarid1c* expression and associated active histone marks at its 5′end are high in P19 neurons, indicating that JARID1C demethylase plays an important role in differentiated neuronal cells. We found that XX mice expressed *Jarid1c* more highly than XY mice, independent of their gonadal types (testes versus ovaries). This increased expression in XX mice is consistent with *Jarid1c* escape from X inactivation and is not compensated by expression from the Y-linked paralogue *Jarid1d*, which is expressed at a very low level compared to the X paralogue in P19 cells. Our observations suggest that sex-specific expression of *Jarid1c* may contribute to sex differences in brain function.

## Introduction

Mutations in *JARID1C* are one of the leading causes of X-linked mental retardation [1], [2]. Mouse *Jarid1c* and human *JARID1C* encode a highly conserved JmjC-domain protein that catalyzes the removal of methyl groups from tri- or di-methylated lysine 4 on histone H3 [3]–[5]. Since this histone modification is associated with enhanced gene activity, demethylation of H3 lysine 4 by JARID1C leads to transcriptional repression [6]. In particular, neuronal specific genes are repressed in stem cells and non-neural tissues in part due to JARID1C-mediated H3K4 demethylation at promoter sequences of these genes [7]. Besides its role in neuronal differentiation, JARID1C is involved in neuronal cell death and dendritic growth [3]. To better understand the pathophysiology of mental retardation and increased aggression caused by *JARID1C* mutations [1], [2], [8], it is important to determine where JARID1C accumulates in the brain. In the present study we determined the pattern of expression of *Jarid1c* in mouse brain sections by *in situ* hybridization.

In human and in mouse, the *JARID1C/Jarid1c* gene escapes X inactivation, i.e. it is expressed from both alleles in females [9], [10]. Thus, it is not surprising that *Jarid1c* expression is higher in brains from adult females compared to males [11]. However, the role of sex hormones such as testosterone and estrogens, which could dramatically affect gene expression, was not investigated in our previous study. To simultaneously examine the effects of sex hormones (male versus female) and of the sex chromosome complement (XY versus XX), we have now used a transgenic mouse model, which consists of four genetically distinct types of mice: XX normal females, XY^−^ females (XY mice sex-reversed by deletion of *Sry*), XY^−^ *Sry* males (XY^−^ mice with an *Sry* transgene to restore the male sex), and XX*Sry* males (XX mice sex-reversed by insertion of an *Sry* transgene) [12], [13].

An important question is whether the sex difference in *Jarid1c* expression is present in all tissues and developmental stages. The reported expression of *Jarid1c* from the inactive X ranges between 20% and 100% of that from the active X chromosome, depending on the tissue [14]–[16]. Furthermore, we have previously shown that *Jarid1c* is transiently silenced on the inactive X chromosome in early development, suggesting that *Jarid1c* may have a similar expression level between the sexes at certain developmental stages [16]. Therefore, we compared *Jarid1c* expression in neonates and adult mice. Since the higher expression of *Jarid1c* in females could theoretically be compensated for in males by expression from the Y-linked paralogue, *Jarid1d*, we also examined expression of this gene. The two paralogues are highly similar in nucleotide and amino acid sequence and both function as histone demethylases [5], [10]. However, it is plausible that the two paralogues differ in their expression patterns across tissues as a result of differences in their developmental regulation. For instance, *Utx* and *Uty*, another X–Y paralogous gene pair, appear to be differentially regulated and expressed in the brain [17]. We tested this possibility by in situ hybridization to brain sections. In addition, quantitative RT-PCR was done in male P19 embryonic carcinoma (EC) cells (hereafter P19 stem cells) that can be differentiated into neurons. This study was extended to four additional X/Y gene pairs. Transcriptional regulation of genes is modulated by histone modifications, DNA methylation, and non-coding RNA binding [18]. Active histone marks include histone H3 and H4 acetylation at lysine residues (H3ac and H4ac), and H3 di- or tri-methylation at lysine 4 (H3K4me2 or H3K4me3; [6], [19]). Using chromatin immunoprecipitation (ChIP), we tested three active chromatin modifications at the 5′end of *Jarid1c* and *Jarid1d* in both P19 stem cells and P19 neurons.

Our results demonstrate that higher expression of *Jarid1c* in adult mouse brain is associated with the presence of two X chromosomes, regardless of phenotypic sex. We showed that the female bias in *Jarid1c* expression was apparently not present in neonatal brain and adult liver, suggesting that it may be tissue- and developmental stage-specific. We determined that expression from the Y-linked paralogue *Jarid1d* is very low in P19 stem cells and in differentiated neurons, suggesting that it does not compensate for the difference in *Jarid1c* levels between males and females in these cell types. The two paralogous sequences were occupied by differently modified histones, consistent with their transcription level.

## Results

### *Jarid1c* is expressed in specific brain regions

**Figure 1:**
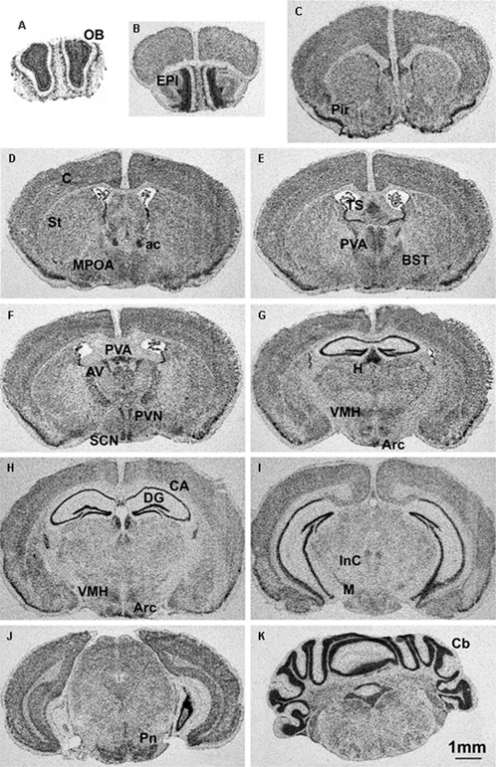
Expression of *Jarid1c* in adult mouse brain using *in situ* hybridization with riboprobes. Examples of sections from an adult female brain are shown. Specific brain regions with higher expression levels are the olfactory bulb (OB), piriform cortex (Pir), habenula (H), hypothalamus such as suprachiasmatic nucleus (SCN), ventromedial hypothalamic nucleus (VMH) and arcuate nucleus (Arc), hippocampus (CA), and cerebellum (Cb). ac: anterior commissure; Arc: arcuate nucleus; AV: anteroventral thalamic nucleus; BST: bed nucleus of the stria terminalis; C: cortex; CA: CA1 and CA3 subfields of hippocampus; DG: dentate gyrus; EPI: external plexiform layer of olfactory bulb; InC: interstitial nucleus Cajal; M: mammillary nucleus; MPOA: medial preoptic area; Pir: piriform cortex; Pn: pontine nuclei; PVA: paraventricular thalamic nucleus; PVN: paraventricular nucleus; St: striatum; TS: Triangular septal nucleus.

Using *in situ* hybridization with an antisense riboprobe, *Jarid1c* mRNA was detected throughout brain sections from male and female adult mice. Higher expression levels relative to surrounding areas were found in the olfactory bulb, piriform cortex, habenula, hypothalamus (such as paraventricular nucleus [PVN], suprachiasmatic nucleus [SCN], ventromedial nucleus [VMH] and arcuate nucleus), hippocampus, and cerebellum (Fig. 1), all of which are brain regions with high cell densities. In addition, *Jarid1c* mRNA was detected in the triangular septal nucleus, anterior paraventricular thalamic nucleus, bed nucleus of the stria terminalis (BST), anteroventral thalamic nucleus, interstitial nucleus of Cajal, mammallary nuclei, and pontine nuclei. In males, the Y-linked paralogue *Jarid1d* was transcribed in similar brain regions as *Jarid1c*, but at relatively lower levels, as revealed with *in situ* hybridization (data not shown), implying that the two paralogous genes may be similarly regulated across brain regions.

### The female bias in *Jarid1c* expression in brain depends on the sex chromosome complement

**Figure 2:**
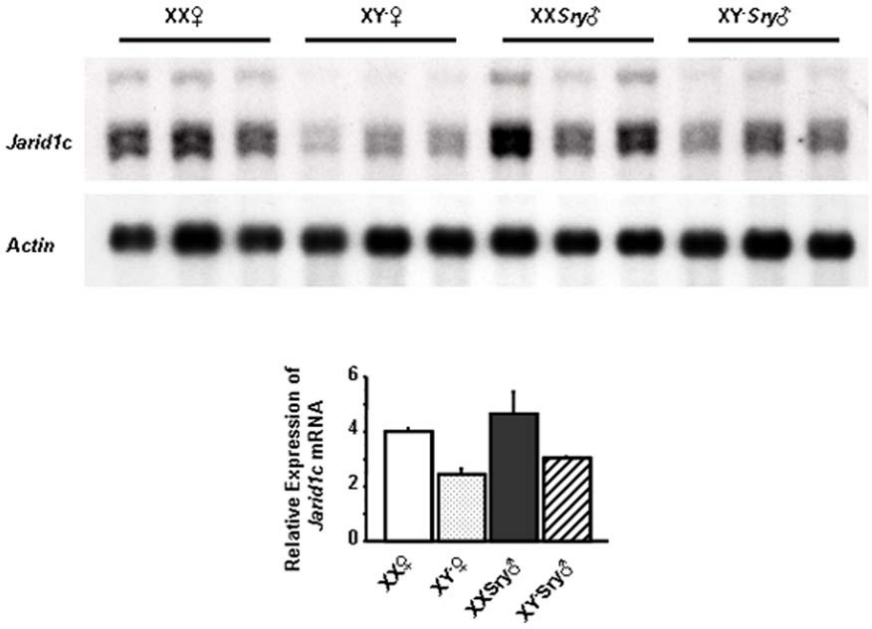
Jarid1c expression level depends on the number of X chromosomes, not on gonadal sex. Example of a northern blot containing mRNA from brains of XX females, XY^−^ females, XX*Sry* males and XY^−^ *Sry* males hybridized to a *Jarid1c* probe. Hybridization to a probe for *Actb (Actin)* was used as a loading control. The graph below shows the relative expression of *Jarid1c* versus *Actb* (β-actin) quantified by densitometry. XX*Sry* males and XX females had significantly higher *Jarid1c* expression than XY^−^ *Sry* males and XY^−^ females, with no difference between mice possessing testes or ovaries.

We have previously shown that *Jarid1c* is expressed more highly in brains of female mice compared to male mice [11]. To test whether this difference was influenced by the sex chromosomes, sex hormones, or both, we compared *Jarid1c* expression in brains from adult XX females, XY^−^ females, XX*Sry* males and XY^−^ *Sry* males by Northern blot analyses. Three independent samples, each from two pooled adult brain samples were analyzed for each genotype. *Jarid1c* expression was normalized against that of *Actb* (β-actin gene) A two-way ANOVA revealed a main effect of the sex chromosome complement, with XX mice having higher levels than XY mice (Fig. 2; F[1], [8] = 7.038; p<.05). There was no main effect of gonadal sex (male versus female) and no significant interaction. We conclude that the higher level of *Jarid1c* expression in females is mainly due to the presence of two active copies of the gene, consistent with escape from X inactivation.

**Figure 3:**
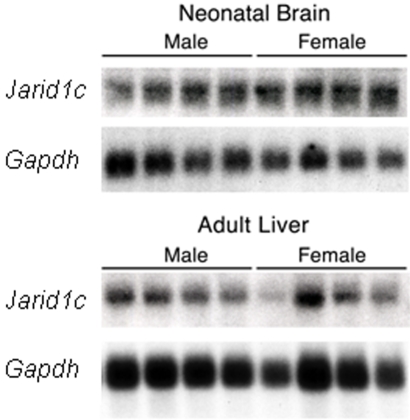
Expression of *Jarid1c* in adult liver and neonatal brain is similar between males and females. Example of a northern blot containing mRNA from four neonatal brain samples and four liver samples from males and females hybridized to a probe for *Jarid1c*. Hybridization to a probe for *Gapdh* was used as a loading control.

We also tested *Jarid1c* expression in neonatal brains and adult livers, but found no significant sex difference in either tissue (p>.05 in both cases), suggesting the sex difference in *Jarid1c* expression found in adult brain might be tissue- and age-specific (Fig. 3). There were, however, noticeable variations among females in both neonatal brains and adult livers and some females did show a higher level of *Jarid1c* mRNA than males (Fig. 3). This is possibly caused by individual difference in transcription of escapee genes from the inactive X chromosome. Similar observations have shown that in women the level of expression of escape genes from the inactive X chromosome varies between individuals and between tissues of the same individual [20].

### *Jarid1c* expression is higher than that of *Jarid1d* in P19 neurons

Using quantitative RT-PCR, expression levels of *Jarid1c* and *Jarid1d* were measured in P19 stem cells and differentiated neurons. Three samples of each type were measured in duplicate. *Jarid1c* expression was about 280-fold higher than that of *Jarid1d* both in undifferentiated and neuron-differentiated P19 cells (Table 1). Four other X–Y gene pairs, *Ddx3x/y*, *Eif2s3x/y*, *Usp9x/y*, and *Utx/y* were also tested for comparison. Interestingly, all five X-linked genes, *Jarid1c*, *Ddx3x*, *Eif2s3x*, *Usp9x*, and *Utx*, were expressed consistently higher than their Y-linked paralogues, both in P19 stem cells and neurons (Table 1). Ratios between X-linked and Y-linked gene expression levels ranged between 150 and 1000 (Table 1).

**Table 1:** Expression (mean±SEM) of X- and Y-linked paralogues in P19 stem cells and neurons.

Gene	Cell type	X-linked paralogue	Y-linked paralogue	X / Y expression ratio
*Jarid1c – Jarid1d*	stem	28±3	0.1±0.006	280
	neuron	83±15	0.3±0.06	277
*Ddx3x – Ddx3y*	stem	826±134	2.0±0.2	413
	neuron	1907±262	3.5±0.5	545
*Eif2s3x – Eif2s3y*	stem	676±67	4.5±0.1	150
	neuron	1349±242	9.1±1.1	148
*Usp9x – Usp9y*	stem	30±2	0.09±0.01	333
	neuron	137±23	0.3±0.09	457
*Utx – Uty*	stem	4.1±0.1	0.004±0.001	1025
	neuron	9.7±2.2	0.01±0.004	970

### Chromatin remodeling at *Jarid1c* in P19 neural differentiation

**Figure 4:**
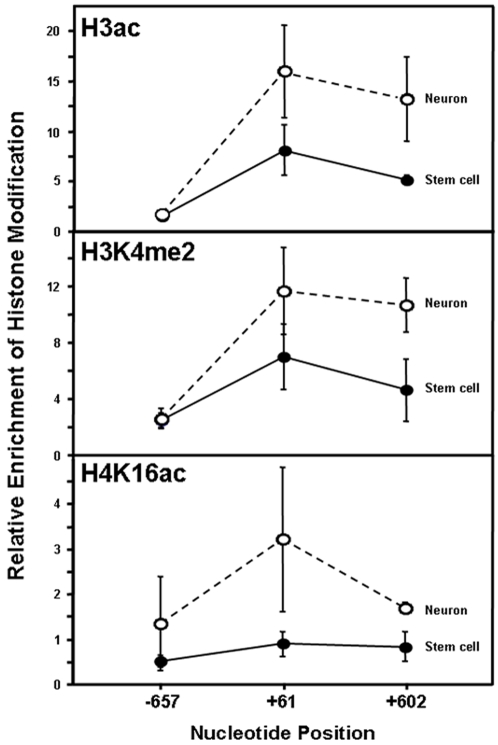
Expression of *Jarid1c* in P19 neurons is associated with active chromatin marks. Ratios between the bound and input chromatin fractions from P19 stem cells (solid lines) and P19 neurons (dashed line) were measured by Q-PCR at three sites around *Jarid1c* transcription start site indicated on the Y axis. Histone H3 acetylation at lysine 9 and lysine 14 (H3ac), H3 di-methylation at lysine 4 (H3K4me2) and to a lesser extent H4 acetylation at lysine 16 were enriched at the 5′ end *of Jarid1c* especially in P19 neurons.

To examine the chromatin structure of *Jarid1c/Jarid1d* in P19 cells before and after differentiation, three sites located at the 5′ end of *Jarid1c* (nt −657 to −465, nt67 to 195, and nt602 to 702) were examined for H3 acetylation, H3 di-methylation at lysine 4, and H4 acetylation at lysine 16 by ChIP. Each of these histone modifications, known to be associated with gene activation, was analyzed with two-way ANOVAs to test the effects of cell types and DNA sites. One site at the 5′end of *Jarid1d* (nt −790 to −594) was examined for these three histone marks. For *Jarid1c* all three histone marks showed a significant enrichment in P19 neurons relative to stem cells (p<0.05) (Fig. 4). We were unable to detect any enrichment in these three histone marks on *Jarid1d* sequences, indicating the absence of these modifications on the Y-linked sequence. We further performed a ChIP-on-chip analysis by hybridization of the ChIP fraction from P19 neurons bound to an antibody to histone H4 acetylated at lysine 16 to a mouse tiling array (MM8_tiling set38) representing the second half of the mouse X and the entire Y chromosome. The array data showed a significantly higher accumulation of H4K16ac along *Jarid1c* gene body compared to *Jarid1d*, the latter showing signals no higher than background (data not shown).

## Discussion

The high expression of *Jarid1c* that we found in the mouse hippocampus is consistent with the cognitive defects in human patients with *JARID1C* mutations [1], [2]. Some of these patients also exhibit elevated aggression [2], an emotion governed by certain brain regions including the bed nucleus of the stria terminalis (BST) [21]. Interestingly, we detected relatively high levels of *Jarid1c* mRNA in the corresponding regions of the mouse brain, including the BST and anterior hypothalamus. These regions have also been implicated in aggressive behavior in rodents [21]. In adult mouse brain, the expression pattern of *Jarid1c* was generally in line with cell densities, i.e. brain regions with higher cell density showed higher levels of hybridization signals. However, some areas (including triangular septal nucleus, anterior paraventricular thalamic nucleus, BST, anteroventral thalamic nucleus, interstitial nucleus of Cajal, mammallary nuclei, and pontine nuclei) showed high expression despite low neuron densities, suggesting specifically enhanced expression in these cell types. We are currently carrying out detailed analyses to locate and quantify the transcripts inside specific cellular compartments in males and females.

*Jarid1c* encodes a histone demethylase specific for histone H3 where it converts tri-methylated lysine 4 to di- and mono-methylated forms [3], [7]. This activity results in gene repression by removal of the active epigenetic mark. Although the complete list of genes targeted by JARID1C is not fully established, one group of neuronal genes that shares similar motifs at their promoter is regulated by this demethylase [7]. These genes are stably repressed in stem cells and non-neural tissues following binding of the REST complex that consists of several proteins including REST, JARID1C and other chromatin modifying enzymes [7]. Surprisingly, we found that *Jarid1c* was highly expressed in P19 differentiated neurons, a finding seemingly contradictory to JARID1C's role as a powerful repressor of neuronal specific genes [7]. Our observations, which were obtained on fully differentiated neurons, support the hypothesis that JARID1C plays an additional role in neurite development. In a previous study a *Jarid1c* knockdown in cultured cerebellar neurons led to shorter neurites [3].

Using a mouse model to distinguish the effects of steroid hormones from those of the sex chromosome complement [22] we found that XX mice had a higher level of *Jarid1c* than XY mice, irrespective of whether they were phenotypic males with testes or females with ovaries. These findings are best explained by the fact that XX mice have two actively transcribed copies of *Jarid1c* due to escape from X inactivation. Although *Jarid1c* is transcribed from both copies in a XX mouse, the expression from the inactive X chromosome is often lower relative to the active X chromosome [14]–[16]. Although the parsimonious explanation for the sex difference in *Jarid1c* expression is that XX females have two actively transcribed copies relative to males having one copy, it is possible that the transcriptional activity of *Jarid1c* differs between one of the two copies in females and the single copy in males. For instance, there is a possible gonadal steroid effect on *Jarid1c* expression since we have not tested by manipulating steroid hormones' levels in gonadectomized mice. Moreover, it is also possible that a Y-linked factor, such as JARID1D suppresses the expression of *Jarid1c* in XY mice. It is not certain whether the sex differences we observed for *Jarid1c* mRNA will necessarily result in a corresponding difference at the protein level. Indeed, *Eif2s3x*, another gene that escapes X inactivation in humans and mice, displays sex specific expression at the mRNA level, but not at the protein level [23].

Expression of Y-linked paralogues in males could theoretically compensate for the high expression of genes that escape X inactivation in females [24]. However, we found that expression of all five mouse X-linked genes examined here was consistently higher – up to a thousand fold – than that of their Y paralogues. Thus, it appears that the Y-linked paralogues did not compensate for higher female-specific expression in these cultured cells. This is consistent with our previous observation in mouse brain that the summed expression of X- and Y-linked paralogues in males is less abundant than that of the two X paralogues in females, one escaping X inactivation [11]. If our observations are confirmed in humans, this could explain why males who carry *JARID1C* mutations are affected in spite of having a normal copy of the Y-linked *JARID1D* gene. Alternatively, JARID1D, despite an apparently similar function as a histone demethylase [25], may have a different role from JARID1C. Among the five mouse X–Y gene pairs compared in our study, two (*Utx/Uty*, *Usp9x/Usp9y*) have a Y paralogue with a separate function from the X-linked paralogue [17], [26]–[28], which is consistent with the differential expression of the paralogues in P19 cells. The lower expression of all Y paralogues may be due to their location in the generally heterochromatic Y chromosome and/or to the up-regulation of the active X chromosome [29].

Gene expression is tightly regulated by histone modifications. We found that three active histone marks, H3 lysine 4 di-methylation, H3 acetylation, and H4 acetylation at lysine 16, were enriched at the 5′end of *Jarid1c* in P19 neurons compared to stem cells, which suggests a different chromatin conformation in neurons. Acetylation of histone H4 at lysine 16, which is usually enhanced throughout the body and 3′end of expressed genes [30] was present along *Jarid1c* but absent on *Jarid1d*, as expected given the difference in expression of the paralogues. It will be interesting to further characterize changes in histone modifications at the *Jarid1c*/*Jarid1d* sequence in P19 neuronal differentiation, particularly in terms of repressive marks.

In summary, we found that *Jarid1c* was expressed in specific brain regions in adult mice and was up-regulated in mice with two X chromosomes versus those with one X chromosome. Expression of the Y paralogue *Jarid1d* did not appear to compensate for the female bias. The expression patterns and differences in *Jarid1c* expression in brain between males and females may lead to sex differences in specific behavior, possibly including aggression, which will need to be further investigated.

## Materials and Methods

### Animals

Procedures for mouse use were approved by the UCLA Chancellor's Animal Research Committee. Mice were bred from stocks obtained from Jackson Laboratories (C57BL/6J) or as a gift (MF1 mice) from Dr. P. Burgoyne (MRC National Institute for Medical Research, London). Conditions of mouse husbandry and tissue collection and the breeding paradigm to generate sex-reversed and control mice were previously described [11]. The four core genotypes included XX normal females, XY^−^ females (XY mice sex-reversed by deletion of *Sry*), XY^−^ *Sry* males (XY^−^ mice with an *Sry* transgene to restore the male sex), and XX*Sry* males (XX mice sex-reversed by insertion of an *Sry* transgene). All tissues were collected from BL/6 mice except in the Northern analysis of the four core mice which were from MF1 mice. Adult tissues were normally harvested from 8–10 months old mice, with the exception of the four-core mouse brains, which were from 12–14 months old animals.

### Cell culture and neuronal induction

P19 EC cells were cultured in DMEM medium containing 10% fetal bovine serum. Neural differentiation was initiated by plating cells in medium containing 0.3 µM retinoic acid (RA; Sigma, St Louis, MO) in non-adhesive Petri dishes to promote the formation of aggregates. After a 4-day exposure to RA, aggregates were dispersed with trypsin (Invitrogen, Carlsbad, CA) and re-plated on cell culture dishes. Cytosine arabinoside (Ara-C; Sigma) was then added to the medium to inhibit proliferation of non-neuronal cells and to select for neurons, which were differentiated by day 6. Cells were collected on day 10 for mRNA and histone modification analyses. Three independent samples were tested for each cell type.

### In situ hybridization

*In situ* hybridization of brain sections was carried out as described in [17], [23]. The riboprobes were transcribed from linearized plasmids containing either a *Jarid1c* or *Jarid1d* cDNA insert. The *Jarid1c* cDNA was an IMAGE clone (clone ID: 6841578; Invitrogen) that contained a 473 bp long insert starting at position bp 4273 of the GenBank sequence AF127245. The *Jarid1d* riboprobe was transcribed from a cDNA clone that contained a 192bp PCR product (bp 1165–1356 of sequence NM_011419) using a pCRScript kit (Stratagene, La Jolla, CA). The specificity of the *Jarid1c* and *Jarid1d* antisense riboprobes was verified with Northern blots [11]. When a sense strand riboprobe was used, no hybridization signal was detected (data not shown).

### Northern blots

Northern blot hybridization was done as described previously [11]. The template for synthesis of the *Jarid1c* probe was a 234 bp RT-PCR product (bp 1109–1342 of sequence NM_013668). Quantification of band intensity was done using *Gapdh* as a control [11]. *Actb* mRNA was also measured as a loading reference, especially in cases when signals for *Gapdh* mRNA appeared to be saturated (Fig. 2). The two reference genes led to similar results in terms of expression of genes of interests between groups.

### Quantitative RT-PCR

Total RNA isolated with an RNeasy kit (QIAGEN) was reverse-transcribed using a first-strand synthesis kit (Invitrogen). Expression of X- and Y-linked paralogues in P19 stem cells and neurons was determined on a LightCycler system (Roche, Indianapolis, IN). Forward and reverse primer sequences were obtained from the Primer Bank website (http://pga.mgh.harvard.edu/primerbank/; Supplementary Table S1). Expression of *Gapdh* was used as a reference. PCR measurements were repeated at least twice. Standard curves based on serial dilutions of samples were established to correct for differences in efficiencies between primers. The specificity of each primer set was confirmed by alignment of dissociation curves. Expression was compared between X–Y paralogues using a paired t-test in six P19 samples, i.e. for each sample, the X and Y paralogues were compared as a pair. For each gene pair, comparison was made across three undifferentiated and three P19 neuron samples.

### ChIP assays

Chromatin was extracted from three P19 stem cell samples and three P19 neuron samples following the manufacturer's instructions (EZ-ChIP kit; Upstate Biotechnology, Charlottesville, VA). Briefly, formaldehyde-fixed chromatin was incubated for 15 hr at 4°C with 5 µg of antibody (anti-acetylated H3 at lysine 9 and lysine 14, anti-dimethylated H3 at lysine 4, anti-acetylated H4 at lysine 16, Upstate Biotechnology). After a second incubation with Protein-A Sepharose beads (Amersham, Piscataway, NJ), bound DNA was eluted and purified. Real-time PCR quantification of enrichment in histone modifications was done using a Roche LightCycler using primers designed to test three *Jarid1c* sites (Supplementary Table S1). Enrichment for each histone mark was normalized against the input (5% of the amount of chromatin used in each antibody-mediated selection). As a negative control, instead of histone antibodies, normal rabbit serum was incubated with chromatin samples which resulted in barely detectable enrichment. Histone modifications on *Jarid1c* sequences were compared between P19 stem cells and neurons by a 2-way ANOVA with cell type and histone modification as factors. DNA from chromatin fractions obtained for histone H4 acetylation at lysine 16 was also submitted to Nimblegen for array analysis using the manufacturer conditions. The mouse tiling array (2006-07-17-MM8 tiling_set38), which covers the second half of the X chromosome (ChrX:93,883,966- 165,556,020, UCSC mouse genome Feb 2006 assembly) and the complete Y chromosome, was used in this study to closely compare the H4K16 acetylation pattern between *Jarid1c* and *Jarid1d* present on the same array. Analysis was done using the manufacturer software.

## Supporting Information

Table S1 PCR primer sequences.
(0.04 MB DOC)
