# Supplementary material for: Sex-Specific Expression of the X-Linked Histone Demethylase Gene Jarid1c in Brain
Source: PLoS One. 2008 Jul 2;3(7):e2553. doi: 10.1371/journal.pone.0002553 (PMC2438472; doi:10.1371/journal.pone.0002553)
Supplement: Table S1 — PCR primer sequences. (0.04 MB DOC) [file pone.0002553.s001.doc]

Supplementary Table S1. PCR primer sequences.

|  | **Forward Primer** | **Reverse Primer** |
| --- | --- | --- |
| *Jaird1c* | GAG GCC CAG ACA GAG TGA AA | TTG GGA TCT TTA AGG ATG AGC C |
| *Jarid1d* | AGT TGT TCG TAC AAA CCA GTG T | CTG GCG TCC AAC AGG TAG C |
| *Ddx3x* | CAG AGT GGA GGA AGT ACA GCA | TCA CCC CGT GAT CCA AAA CTG |
| *Ddx3y* | GGG TCT GTG ATA AGG ACA GTT CA | CAC GAC CAC CAA TAC CAT CAT AG |
| *Eif2s3x* | GGT GAG GGT GGA GTG ACT CT | TTC CCA TGA GCT ACG TGA CCA |
| *Eif2s3y* | AAA GCC ATT TCT GGT GTT CAC A | GGT CGA GGA CAA CTT GAG TCA T |
| *Usp9x* | TCC AAC AGA ATC AGA CTT CAT CG | TGG AAA TGC AGG TTC CTC ATC T |
| *Usp9y* | CAG CCT TCT TTC CAA CAG AAC C | CTT TGC CGG GTC AGT ATG AGG |
| *Utx* | AAG GCT GTT CGC TGC TAC G | GGA TCG ACA TAA AGC ACC TCC |
| *Uty* | GTT TTG TGG CAT GGG AGG ATA | GAT GGC ACT GTC TCA GGT GG |
| *Gapdh* | AGA GAG AGG CCC TCA GTT GCT | TTG TGA GGG AGA TGC TCA GTG T |
| *Jarid1c* -465 | GGG AGT GAG TTT CCT TCT T | AAT GTC CTT TGC TGC TTC TCT C |
| *Jarid1c* 67 | AAG GTG TGG AAG AAA CGG AAC | GAG TCT AGG CCC TAA GCA GGA |
| *Jarid1c* 602 | TCC TCG TTT TGT GGC ATA CA | CCA GCC CAT TAA GCA TCT GT |
| *Jarid1d* -594 | ACT GGC CTT GAA CTC AGA AAT C | TCT AGT GCC CCA GTA TTT CCA T |
